# Downscaling of Industrial Turbo-Distillation to Laboratory Turbo-Clevenger for Extraction of Essential Oils. Application of Concepts of Green Analytical Chemistry

**DOI:** 10.3390/molecules24152734

**Published:** 2019-07-27

**Authors:** Sandrine Périno, Zoubida Chemat-Djenni, Emmanuel Petitcolas, Christian Giniès, Farid Chemat

**Affiliations:** 1 Green Extraction Team, UMR 408, Avignon University, INRA, F-84000 Avignon, France; 2 Laboratoire d’Analyse Fonctionnelle des Procédés Chimiques (LAFPC), Département Génie des Procédés, Faculté de Technologie, Université Saad Dahlab Blida 1, 09000 Blida, Algeria; 3 MicroNut, UMR 408, INRA, Avignon University, F-84000 Avignon, France

**Keywords:** turbodistillation, downscaling, extraction, essential oil, green analytical chemistry

## Abstract

In the effort of innovation towards green analytical chemistry concepts and considering the six principles of green extraction, the industrial turbodistillation process was downscaled into a laboratory apparatus turbo-Clevenger (TC) for the extraction of essential oils. Turbodistillation is used as an industrial purpose for the extraction of essential oils from hard matrixes such as wood, barks, seeds. In this work, a TC and the conventional technique of hydrodistillation (HD, Clevenger apparatus) are used for the extraction of essential oils from three spices with hard structures (*Illicium verum*, *Schinus terebinthifolius,* and *Cinnamomum cassia*) and are compared. This study shows that the essential oils extracted by TC in 30 min were quantitatively (yield and kinetics profile) and qualitatively (aromatic profile) similar to those obtained using conventional hydrodistillation in 3 h. This process, which gave a reduced extraction time, was perfectly adapted to the extraction of hard matrixes.

## 1. Introduction

In general, an analytical procedure for essential oils from herbs or spices comprises two steps: extraction (steam distillation, hydrodistillation) and analysis (gas chromatography (GC), gas chromatography coupled to mass spectrometry (GC-MS)). Whereas the last step is finished after only 15 to 30 min, extraction takes at least several hours. It is frequently done by prolonged heating and stirring in boiling water using a Clevenger apparatus for hours or days. These shortcomings have led to the consideration of the use of innovative techniques in essential oil extraction that typically use less water and energy, such as ultrasound and microwave [1,2].

Turbodistillation was patented by Martel in 1983 [3] and has been used in several companies (Grasse-France) as an industrial purpose (1000 L to 10 000 L) for the extraction of essential oils from hard matrixes (such as wood, bark, and seeds). The extraction process is the same as hydrodistillation (HD), but a mechanical stirrer breaks down agglomerates and chunks of hard matrices and homogenizes the medium at the same time. It reduces extraction time and energy consumption and allows preventing the degradation of volatile compounds compared to HD. 

Turbodistillation resolves the problem of hard structure, encountered with spice matrices, which makes HD less effective as water has to penetrate a thick physical barrier in order to reach the secretory stores deep inside the structure. Dry milling of hard matrices has been performed, but this extra unit operation generates thermal degradation of the essential oil. Moreover, without stirring, the powdered matrix lies still at the bottom of the reactor, where it can burn, resulting in a degraded essential oil. However, turbodistillation is not a good extraction alternative for soft matrices. Périno-Issartier et al. [4] have compared different extraction processes such as ultrasound, microwave, turbodistillation, steam, and hydrodistillation to obtain essential oils from lavandin. The essential oils are all similar in terms of yields and aromatic profiles and the odor is similar to the original lavender. 

Generally, the authors have reported full studies of processes from laboratory to pilot scale and not the other way around [5]. Filly et al. [6] have proposed a green method for the extraction of essential oil from aromatic herbs without any added solvent or water. This technique, solvent-free microwave extraction (SFME) is a combination of microwave heating and dry distillation. Experiments performed in a 75 L pilot microwave reactor prove the feasibility of SFME upscaling and potential industrial applications.

Downscaling chemistry processes is a growing area of research. Current trends in green analytical chemistry [7,8] involve the development of downscaled instrumentation [9] due to an increase in demand for rapid, cost-effective, and environmentally friendly sample preparation methods prior to analysis: significant shortening of processes with subsequent savings in energy, natural state of the extracts is intact, and minimization of wastes. These past years, there has been a development of downscale sample preparation apparatus that can help to do the in-field extraction, sample preparation, analysis, and data evaluation. In parallel, a portable instrument is defined as “easily” movable, convenient for carrying, and capable of being transferred or adapted in altered circumstances. Downscaling or miniaturization of a procedure can be achieved simply by reducing the dimensions of the systems used in earlier approaches or by developing completely new set-ups or techniques. When compared to conventional systems, miniature systems can perform similar methods with remarkably reduced consumption of plant matrix and solvents, size and power requirements, system costs, with a faster analysis time and massively parallel analysis capability. It is an attractive method for applications requiring in-field rapid assays.

The aim of this work was to adapt the method for the extraction of essential oils from hard matrixes and compare the results with those obtained by conventional techniques, in order to introduce this advantageous alternative in the analytical or production of essential oils in the food, cosmetics, and pharmaceutical industry.

To investigate the potential of the turbo-Clevenger (TC), comparisons were made with conventional hydrodistillation for the extraction of essential oils from Brazilian pepper (dry fruit), Chinese star anise (pericarp), and cinnamon (bark). We intended to make appropriate comparison in terms of extraction time, yield, and aromatic composition.

## 2. Results and Discussion

### 2.1. Extraction Kinetics

**Figure 1:**
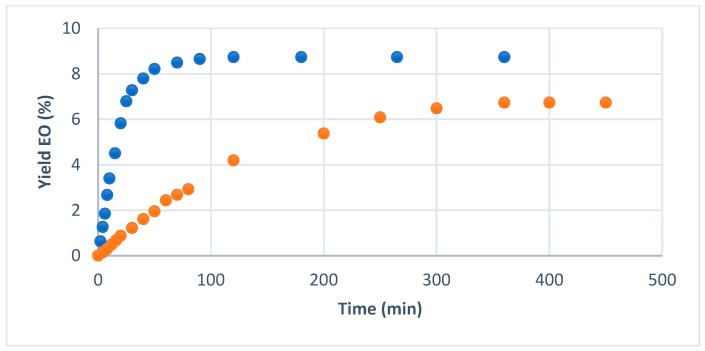
Chinese star anise essential oil yield per 100 g of dry matter (turbo-Clevenger 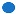 hydrodistillation 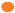).

Patent describes an industrial scale reactor that can contain 800 L of water and approximately 300 kg of dry matter to produce a mass of essential oil ranging few kilograms (40 to 60 kg) over 4 h. Our laboratory-scale TC permitted the extraction of 500 g of dry matter with 2 L of water over 2 h of extraction to produce a few grams of essential oil. The yield is defined as the percentage of weight of essential oil extracted from the initial mass. While reporting the Chinese star anise essential oil extracted volume against the extraction time (Figure 1), three distinct phases in the extraction were distinguished.

These phases are characteristics of natural product extraction, as reported previously [10,11]. The first step from minute 0 to minute 15 for the TC with a very rapid extraction speed, which corresponds to the immediately accessible essential oil, easily transferred to the solvent. This phase occurs from minute 0 to minute 60 in the case of HD. From minute 15 to minute 45 in the TC protocol, a slower steady extraction phenomenon is observed. During this phase, the solvent must penetrate the matter’s hard structure before reaching the essential oil. This phase ranges from minute 60 to minute 300 for HD. Finally, the last phase from minute 45 to the end of the extraction (TC) and from minute 300 to minute 450 (HD) is the plateau that marks the end of the extraction process. The matter has almost been depleted in essential oil. Past 120 min for TC, no further essential oil could be obtained from the matter. This profile is typical to all essential oil extractions [12]. Although the extracted quantity differed from one species to the next, the kinetic profiles were similar for all three spices (data not shown). The main advantage of the TC, in regards to the kinetic of extraction, is that the essential oils were obtained in a dramatically reduced time. We observe here that TC permits us to divide by at least three the extraction time of hard matrices’ essential oils. Whatever the process, yields were at least similar for cinnamon (1.37%) and red pepper (2.22%) essential oils and in accordance with the yield range reported in literature [13,14], but the extraction time was reduced by a factor of three. In the case of Chinese star anise, the yield was improved by a factor of two in 120 min for TC vs HD. The maximum of the yield for HD was obtained in 360 min. The second phase of the extraction kinetic profile is particularly reduced (from 240 min for HD down to 30 min for TC), allowing the final plateau and maximum yield to be obtained very quickly. Turbo-distillation make three actions: detexturing the matrix, allowing better accessibility to glands of essential oil, and at the same time enhancing turbulence for better extraction and enhancement of surface evaporation.

### 2.2. Composition of Essential Oils

All essential oils were similar in all aspects in regards to their sensory properties (appearance, pale color, fragrance). For each spice, essential oils extracted using the different methods were all similar in their composition. The same number of volatile secondary metabolites was found in them (see Table 1). 

Although essential oils contain the same major components, their respective quantities vary more or less according to the extraction technique used.

Moreover, bornyl acetate was extracted with TC, at least twice as much as with the HD process. The minor differences are mostly explained because of the variability of the raw material from batch to batch. Although, while there were still minor, differences in extraction, especially related to components that were absent from the HD extract, can be explained because of the TC’s ability to extract more selectively than the conventional method alone (for example, with Chinese star anise essential oil).

### 2.3. Green Process Assessment

**Figure 2:**
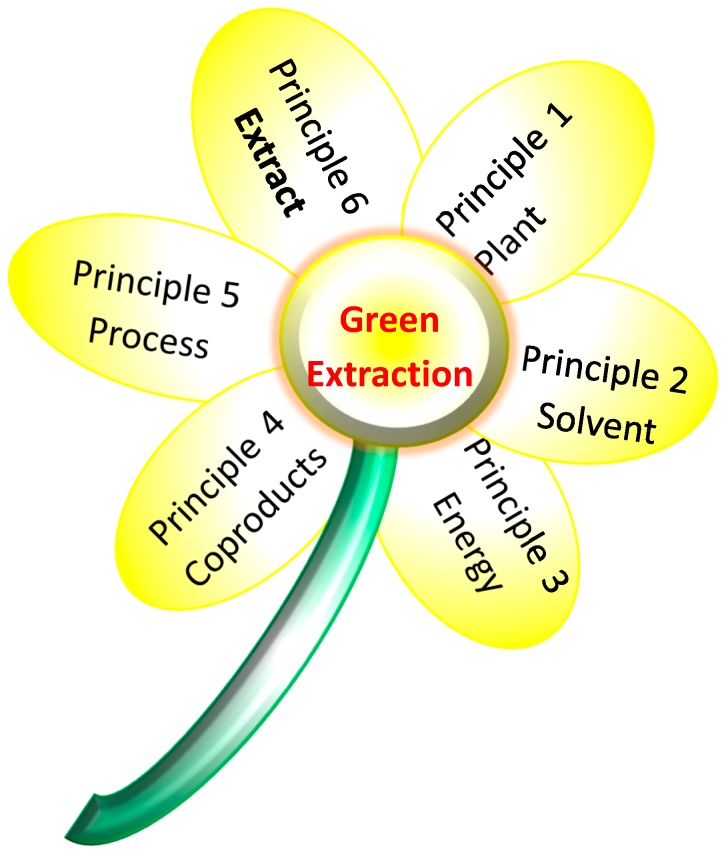
Green process assessment according to green extraction. Principle 1: Innovation by selection of varieties and use of renewable plant resources. Principle 2: Use of alternative solvents and principally water or agro-solvents. Principle 3: Reduce energy consumption by energy recovery and using innovative technologies. Principle 4: Production of co-products instead of waste to include the bio- and agro-refining industry. Principle 5: Reduce unit operations and favor safe, robust, and controlled processes. Principle 6: Aim for a non-denatured and biodegradable extract without contaminants.

Existing extraction technologies are linked to several technological, environmental, and scientific barriers that are generally complex and difficult to overcome, for instance, minimizing energy and toxic solvent consumption, all the while reducing CO_2_ emission and guaranteeing safety and control for both the final product and the technical staff involved. Both the turbo-Clevenger and hydrodistillation processes were evaluated according to the six principles of green extraction that were developed by Chemat et al. [15]. Those principles allow the design of extraction methods that aim to grant a natural and safe extract from well-reasoned sourcing with, instead of waste, a high added value, while reducing the organic solvents, the energy consumption, as well as the process time (Figure 2). It should be noted that principles 1, 2, 4, and 6 were equal for both processes. For principle 3 and 5, there was a dramatic reduction of time and energy.

## 3. Materials and Methods

### 3.1. Plant Material

All three spices (*Illicium verum*, *Schinus terebinthifolius*, and *Cinnamomum cassia*) were bought from local markets (Provence Region, Avignon, France). 

### 3.2. Turbo-Clevenger (TC) Apparatus and Procedure

**Figure 3:**
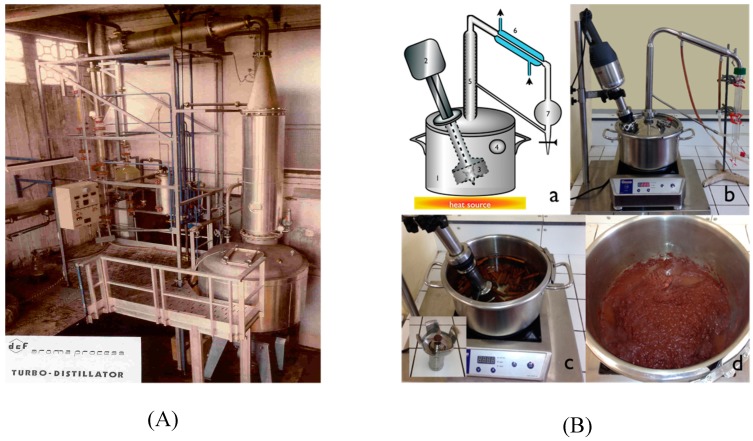
(**A**) Industrial turbo-Clevenger; (**B**) laboratory turbo-Clevenger: (**a**) schematic, (**b**) bench apparatus, (**c**) cinnamon experiment before extraction with mixer detail (insert), (**d**) cinnamon experiment after extraction.

In order to obtain the maximal extraction, TC parameters need to be set to respect two main characteristics. The first characteristic is to grind the agglomerates and biggest chunks of hard matter in order to maximize the contact area for water to penetrate the solid phase. The second parameter is to keep a steady turbulent flow inside the reactor to maximize the evaporation area. The apparatus used in this study is a scaled-down version of industrial turbodistillation reactor (Figure 3A). The vessel (1) (Figure 3Ba) is a classic Alambic reactor (REUS, Grasse, France) and can contain up to 5 L of water and hard matrix (200 to 500 g). We added the adjusted arm that is comprised of a rotor (2) and a reproduction of a turbo shredder (3). The rotor speed is controlled and has to be adjusted so that flow and mixing are adequate for the protocol (turbulent flow and grinding of material). The vessel is heated and temperature is controlled with a thermometer (4). Evaporation under atmospheric pressure occurs at 100 °C, and the vapors charged in essential oils flow through the distillation column (5), enrich the concentration of essential oil, and flow further into the condenser (6) where both the water and essential oil are condensed and drop into the Clevenger apparatus. 

Protocol was as follows: plant material was dipped in 5 L of distilled water (Chinese star anise 200 g, Brazilian red pepper 400 g, cinnamon 300 g). The kinetic was calculated as the first drop of distillate was seen, and extraction was conducted until no more oil was extracted. The essential oil was dried over magnesium sulphate and stored at 4 °C until used.

### 3.3. Hydrodistillation (HD) Apparatus and Procedure

Hydrodistillation was performed using the same reactor type (REUS, Grasse, France) without the use of the adjusted arm (rotor and mixer). All extractions were performed using 5 L of distilled water and the same amount of material (Chinese star anise 200 g, Brazillian red pepper 400 g, cinnamon 300 g). The heat source was set up for the solvent to reach 100 °C. The kinetic was calculated as the first drop of distillate was seen, and extraction was conducted until no more oil was extracted. The essential oil was dried over magnesium sulphate and stored at 4 °C until used.

### 3.4. Gas Chromatography Analysis and Compound Identification

Essential oil composition was determined by gas chromatography coupled to mass spectrometry (GC-MS) using an electronic impact (70 eV) ionisation mode. Analysis was performed on a Hewlett-Packard 6890 coupled to a 5973A mass spectrometer, using HP5MS (30 m × 0.25 mm × 0.25 µm film thickness). GC-MS spectra were obtained using the following conditions: the vector gas was helium, the flow rate for the HP-5MS column was 0.3 mL/min, split-less mode, injection volume, 1 μL; injection temperature, 250 °C; oven temperature program, 60 °C for 8 min then increased at 2 °C/min to 250 °C and held at 250 °C for 15 min. Identification of the components was based on computer matching against commercial libraries (Wiley, MassFinder 2.1 Library, and NIST98), laboratory mass spectra libraries built up from pure substances, and MS literature data combined with the comparison of GC retention indices (RI) on apolar and polar column. RI values were calculated with the help of a series of linear alkanes C_6_–C_26_ on an HP5MS column). Compounds available in the laboratory were confirmed by external standard compound co-injection. Each sample was analyzed three times, and the RI was calculated as the average of the three experiments.

## 4. Conclusions

The essential oils of three spices, Chinese star Anise, Brazilian pepper, and cinnamon, obtained with a turbo-clevenger apparatus were compared against conventional laboratory-scaled hydrodistillation. Although there were no major changes in chemical composition, improvements are reported here regarding the effectiveness of this apparatus. It allows the obtaining of essential oils in similar quantities/yields or even higher. The extraction time was divided by at least three compared to the conventional hydrodistillation process. The energy consumption was consequently dramatically reduced as well. The apparatus can then be used in laboratory to measure kinetics of extraction and to produce small quantities of quality essential oils that can be analyzed immediately. 

## Figures and Tables

**Table 1:** Chemical compositions of three essential oils obtained by turbo-Clevenger (TC) and hydrodistillation (HD).

N°.	Compounds ^a^	RI ^b^	Brazilian Pepper		Cinnamon		Chinese Star Anise	
		HP5MS	HD (%)	TC (%)	HD (%)	TC (%)	HD (%)	TC (%)
	**Monoterpenes**		**86.25 ± 0.05**	**87.06 ± 0.05**	**0.36 ± 0.01**	**0.99 ± 0.02**	**2.2 ± 0.04**	**4 ± 0.02**
1	α-pinene	928	7.83 ± 0.11	8.67 ± 0.12	0.12 ± 0.01	0.41 ± 0.02	0.10 ± 0.08	0.30 ± 0.05
2	Sabinene	966	1.33 ± 0.01	1.39 ± 0.01	-	-	tr.	0.10 ± 0.01
3	β-pinene	970	0.57 ± 0.01	0.73 ± 0.01	0.11 ± 0.01	0.32 ± 0.01	tr.	0.10 ± 0.01
4	β-myrcene	987	4.24 ± 0.02	4.50 ± 0.02	tr.	tr.	0.10 ± 0.01	0.20 ± 0.01
5	α-phellandrene	1005	54.0 ± 0.24	54.25 ± 0.19	-	-	0.10 ± 0.03	0.10 ± 0.01
6	p-cymene	1020	1.65 ± 0.01	1.59 ± 0.01	tr.	tr.	0.10 ± 0.06	tr.
7	Limonene + β-phellandrene	1024	15.48 ± 0.04	14.87 ± 0.02	0.13 ± 0.02	0.26 ± 0.02	1.80 ± 0.01	3.10 ± 0.02
8	α-terpinolene	1080	1.15 ± 0.01	1.06 ± 0.01	-	-	tr.	0.10 ± 0.01
	**Oxygenated monoterpenes**		**0.68 ± 0.01**	**0.28 ± 0.01**	**6.02 ± 0.02**	**8.11 ± 0.03**	**1.1 ± 0.01**	**0.8 ± 0.01**
9	Eucalyptol	1026	-	-	0.85 ± 0.01	1.19 ± 0.03	0.20 ± 0.01	0.10 ± 0.01
10	Linalool	1099	0.47±0.01	0.18±0.01	0.25 ± 0.01	0.3 ± 0.01	0.70 ± 0.02	0.60 ± 0.03
11	4-terpineol	1173	0.21±0.01	0.10±0.01	0.40 ± 0.01	0.37 ± 0.01	0.20 ± 0.01	0.10 ± 0.01
12	α-terpineol	1190	tr.	tr.	0.58 ± 0.01	0.52 ± 0.01	-	-
13	Bornyl acetate	1276	tr.	tr.	0.37 ± 0.04	0.79 ± 0.06	-	-
14	Cinnamyl acetate	1445	-	-	3.57 ± 0.02	4.94 ± 0.03	-	-
	**Sesquiterpenes**		**5.6 ± 0.03**	**6.13 ± 0.02**	**-**	**-**	**-**	**0.5 ± 0.02**
15	trans β-caryophyllene	1405	1.50 ± 0.02	1.69 ± 0.01	-	-	-	-
16	δ-elemene	1326	0.15 ± 0.01	0.15 ± 0.01	-	-	-	-
17	(E)-α-bergamoten	1424	0.13 ± 0.01	0.11 ± 0.01	-	-	tr.	0.50 ± 0.02
18	Germacrene D	1467	2.27 ± 0.07	2.30 ± 0.06	-	-	-	-
19	δ-cadinene	1507	0.68 ± 0.02	1.08 ± 0.03	tr.	tr.	-	-
20	γ-elemene	1541	0.87 ± 0.06	0.80 ± 0.01	-	-	-	-
	**Other oxygenated compounds**		**2.81 ± 0.06**	**2.52±0.03**	**93.15 ± 0.06**	**89.37 ± 0.02**	**94.9 ± 0.07**	**89.7 ± 0.08**
21	Benzaldehyde	960	-	-	0.11 ± 0.01	0.34 ± 0.02	-	-
22	3-phenylpropanal	1160	-	-	0.48 ± 0.03	0.36 ± 0.02	-	-
23	Estragole	1194	-	-	-	-	5.30 ± 0.03	4.10 ± 0.06
24	Cis-cinnamaldehyde	1214	-	-	0.5 ± 0.02	0.5 ± 0.02	-	-
25	Anisaldehyde	1247	-	-	-	-	2.60 ± 0.04	0.20 ± 0.01
26	trans-cinnamaldehyde	1266	-	-	91.44 ± 0.25	87.56 ± 0.20	-	-
27	Isoestragole	1288	-	-	-	-	87.00 ± 0.15	85.40 ± 0.18
28	2H-1-benzopyranone	1432	-	-	0.62 ± 0.01	0.61 ± 0.01	-	-
29	Elemol	1540	0.66 ± 0.05	0.52 ± 0.07	-	-	-	-
30	Spathulenol	1564	0.56 ± 0.02	0.57 ± 0.02	-	-	-	-
31	γ-eudesmol	1620	0.34 ± 0.11	0.27 ± 0.01	-	-	-	-
32	β-eudesmol	1644	1.25 ± 0.07	1.16 ± 0.04	-	-	-	-

^a^ Compounds are listed in order of their elution time from an HP5MS column. Compositional values less than 0.1% are denoted as traces (tr). ^b^ RI: retention indices are determined on an HP5MS column.
